# Freshwater organisms potentially useful as biosensors and power-generation mediators in biohybrid robotics

**DOI:** 10.1007/s00422-021-00902-9

**Published:** 2021-11-23

**Authors:** Wiktoria Rajewicz, Donato Romano, Joshua Cherian Varughese, Godfried Jansen Van Vuuren, Alexandre Campo, Ronald Thenius, Thomas Schmickl

**Affiliations:** 1 649 Institute of Biology, Graz, 8010 Austria; 2 University of Graz, Graz, Austria; 3 The BioRobotics Institute, Sant’Anna School of Advanced Studies, Viale Rinaldo Piaggio 34, Pontedera, Pisa 56025 Italy; 4 Department of Excellence in Robotics and AI, Sant’Anna School of Advanced Studies, Pisa, 56127 Italy; 5 Unit of Social Ecology, Universit é Libre de Bruxelles, Campus Plaine, Boulevard duTriomphe, CP 231, 1050 Bruxelles, Belgium

**Keywords:** Biomonitoring, Biohybrid, Animal–Robot interaction, Biorobotics

## Abstract

Facing the threat of rapidly worsening water quality, there is an urgent need to develop novel approaches of monitoring its global supplies and early detection of environmental fluctuations. Global warming, urban growth and other factors have threatened not only the freshwater supply but also the well-being of many species inhabiting it. Traditionally, laboratory-based studies can be both time and money consuming and so, the development of a real-time, continuous monitoring method has proven necessary. The use of autonomous, self-actualizing entities became an efficient way of monitoring the environment. The Microbial Fuel Cells (MFC) will be investigated as an alternative energy source to allow for these entities to self-actualize. This concept has been improved with the use of various lifeforms in the role of biosensors in a structure called ”biohybrid” which we aim to develop further within the framework of project Robocoenosis relying on animal-robot interaction. We introduce a novel concept of a fully autonomous biohybrid agent with various lifeforms in the role of biosensors. Herein, we identify most promising organisms in the context of underwater robotics, among others *Dreissena polymorpha*, *Anodonta cygnaea*, *Daphnia* sp. and various algae. Special focus is placed on the ”ecosystem hacking” based on their interaction with the electronic parts. This project uses Austrian lakes of various trophic levels (Millstättersee, Hallstättersee and Neusiedlersee) as case studies and as a ”proof of concept”.

## Introduction

Climate change and water pollution have been a topic of growing concern for many decades now. Water is an essential resource for life, economy and climate regulation. Overpopulation with rising industrialism, farming and other sources of waste, have led to an immense water pollution problem (Damania et al. 2019). Water monitoring plays a crucial role in protecting the lifeforms inhabiting it and preserving the balance of the ecosystem. Environmental studies are dominated by *ex situ* investigations including sampling and laboratory analysis (Martins et al. 2007; Fai et al. 2007; Alexander and Mcmahon 2004). While this approach can give accurate data on specific parameters, it is time, money and labour consuming. This leads to a slower monitoring process on a larger scale making it more difficult to detect sudden changes (natural and anthropological) within a short time frame. A need arose to develop more efficient, affordable and continuous methods of water monitoring. The use of robots has become a subject of interest as it allows obtaining accurate data in a continuous manner with minimal human interference. This is especially useful in non-easily accessible habitats, such as deeper or protected lakes where sampling can be challenging or very restricted.

The project Robocoenosis (Thenius et al. 2021) approaches the concept of long-term, autonomous water monitoring devices with the introduction of ”autonomous biohybrid entities”. Here, a biohybrid is considered a structure that connects biological entities and synthetic materials that allows extracting useful data from said entities (Sun et al. 2020). The use of biohybrids became a dynamic field of science that has found its use in monitoring both aquatic and terrestrial environments (Gutiérrez et al. 2015; AquaDect 2021). Robocoenosis introduces a new approach of ”lifeform in a loop” which allows for further development of self-actuating biohybrid entities for environmental monitoring. Biohybrids allow an early detection of pollutants and other stressors in a safe, noninvasive for the environment way, while allowing the robots to self-actualize. It creates a link into the ecosystem without the need of excessive sampling. The use of biohybrids opens the door to a novel paradigm of ”ecosystem hacking” where the lifeforms and their interaction with the environment are used to detect and/or prevent the collapse of the ecosystem. The ability to detect sudden changes quickly leads to a better understanding of the processes occurring in the ecosystem than what can be achieved with just classical monitoring methods.

Biohybrid robotics has been gaining popularity for many years now. It is often a convenient method to optimize the performance of artificial materials while using already naturally occurring biological processes. Special attention has been paid to the self-actualization of robots using anything from enzymes and single cells to entire muscle tissues used as the robots’ propelling mechanism (Mestre et al. 2021). Projects developing biohybrid robots are dominated by laboratory trials that serve as a proof-of-concept. Small robotic jellyfish propelling through water with the use of rat cardiomyocytes has been developed by (Nawroth et al. (2012)). Lifeforms such as sperm cells, rats, earthworms and frogs have been successfully used as biohybrid actuators in many microrobotics studies (Ricotti et al. 2017). However, the animal-robots interaction is not limited to using lifeforms as the power source. Robots have also been designed to copy and study animal behaviour by inserting the electronics into a living organism in order to stimulate it in a controlled and reproducible way (Romano et al. 2019). Such a behaviour imitation was used to investigate the social hunting mechanisms and kleptoparasitism of an archerfish by constructing a robotic copy of the fish (Brown et al. 2021). This approach has been used to study in detail the physiology and behaviour of many terrestrial and aquatic species, such as geckos, carps, cockroaches and many others. In conclusion, biohybrid robotics have been used in mostly two variations: (1) parts of living organisms being incorporated into a mechanical structure and (2) robotic parts being incorporated into a lifeform (Schwefel et al. 2014; Nawroth et al. 2012). The project Robocoenosis will utilize a different approach of minimizing the impact on the lifeforms and use various methods of pure observation of the animal-robot interaction.

This concept finds its uses in a variety of projects. MusselMonitor uses clams as a first-degree bioindicator. Sensors placed on each valve send an electric current that is measured continuously (AquaDect 2021). Closing of the valves is detected early and treated as a sign of stress which then, sounds the alarm. Another example of this concept is Daphnia Toximeter II which uses *Daphnia* sp. swimming pattern to detect water pollution (Green et al. 2003). The animals are continuously monitored using video image analysis, and an alarm is triggered if their behaviour deviates from normal. While these designs show promising results, they are heavily laboratory-based or are limited to one species. In Robocoenosis, we aim to use a variety of lifeforms to obtain a more well-rounded information on the ecosystem. We plan on taking a step away from the laboratory and move the entirety of monitoring into the field of autonomous robotics. The autonomous biohybrid will provide large amounts of real-time data while being completely environmentally friendly at a low cost.

For this biomonitoring goal, special attention is placed on the lifeform-robot interaction. For optimal results in the early experiments, it is essential to use setups specifically designed for hosting chosen species as to ensure their presence for the duration of the experiments. Lifeforms are to be collected directly beforehand and immediately placed on the biohybrid which will minimize possible stress caused by handling and changing environments. The project also heavily relies on so-called biofouling, meaning the process of accumulation of undesired aquatic organisms, such as mussels, algae or bacteria, on an underwater structure (Uzun et al. 2021). While this is commonly one of the main obstacles in aquatic long-term monitoring, in Robocoenosis, this will be used as one of the methods of incorporating the species naturally occurring in the lake and using them as biosensors.

### Main objectives of the Robocoenosis project

**Fig. 1:**
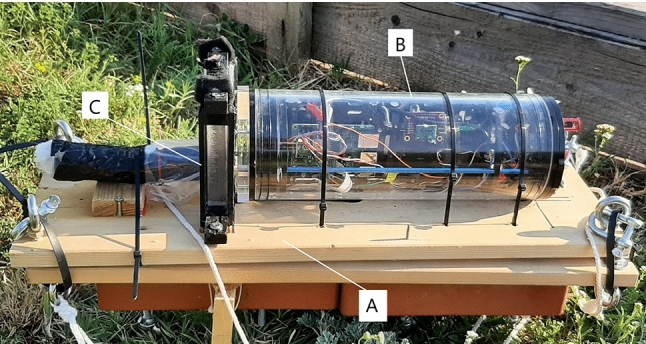
First prototype of the biohybrid entity developed as part of the project Robocoenosis. Various parts will serve different functions and ultimately operate together. **a** Biodegradable frame, **b** Technological elements and mission terminating module C: Organs hosting different bioindicators.

Core features of the biohybrid entity being developed during the project Robocoenosis are presented in the early prototype (Fig. 1). The emphasis is put on the use of lifeforms and biodegradability.

*Biomonitoring* The entities developed will enable continuous, real-time monitoring while being as little invasive as possible. The electronics setup will use lifeforms and their interaction with the environment (here called “ecosystem hacking”) to provide a new form of biomonitoring through identifying potential stressors in the water.

*Biodegradability* Special attention is being given to minimizing the impact of the research on the environment. The entity will comprise of two parts: 1) non-biodegradable module that will be retrieved after finalizing the mission and 2) a degradable module that will remain in the habitat and decompose later on. This will be achieved with the use of paper, wood and other degradable materials. The non-degradable parts will detach themselves and return to the surface after the project/scenario will have been terminated.

*Long-term autonomy* By using energy harvesting and low-power electronics, we ensure the self-actuation of the Robocoenosis biohybrid entity. The use of Microbial Fuel Cells (MFCs) will be tested and improved in the field as a power source, as well as a form of biosensor (measuring the metabolic activity of bacteria).

## Methods

To reach the previously described goals, a thorough investigation was performed to determine the feasibility of various lifeforms in context of autonomous biomonitoring. In this paper, we will outline most promising examples of living organisms (i.e. invertebrates, algae, bacteria) and ways to utilize them for autonomous experimental setups.

Aquatic microorganisms can give valuable information on many different aspects of the environment, such as (Perera and Wattavidanage 2011):

- chemical (i.e. pH, heavy metals, nutrients)
- physical (i.e. temperature, light intensity, depth)
- ecological (i.e. species composition, natural or anthropological changes)

They can be used at various organisation levels ranging from one individual to a whole community structure. The latter proves useful when the state of the environment is evaluated with the use of various indices. These indices can be based on the presence of certain species characteristic for a specific environment, biomass of dominant species and many others, which will be described later on (Heinonen 1980).

### Incorporating lifeforms into the autonomous experimental setups

There are three likely approaches for the automated system to use the lifeforms: (1) forcing the organism onto the platform, (2) inviting it and (3) providing no interaction with the environment. Forcing the lifeform can be done by attaching the organism to the setup before the start of the monitoring process, i.e. by glue or entrapment. This method will ensure the presence of the lifeform and enable predictable response. It does, however, involve handling the animals which can prove stressful and is quite limited in terms of the species variety that can be investigated at one given time. It also involves researching whether the used lifeform is already present in the investigated habitat to prevent introducing foreign species. Second approach involves using a blank platform provided with a variety of substrata designed to host a group of species. This could involve coarse or rock-like surfaces that will enable mussel settlements, providing tube-like structures to encourage larvae development of certain species and others. This method is less invasive and will provide a bigger variety of observable species, however, to some extent, relies on chance. Third method is noninvasive and based solely on observation. Data on presence/absence and/or abundance of certain species can prove useful and cannot be easily predicted and accounted for beforehand; therefore, this will be carried out in addition to the other approaches mentioned above.

### Site description

To test the biohybrid entity, various lakes were chosen representing vastly different habitats: glacial lakes and shallow endorheic lake. Lakes Millstätter See (46$^{\circ }$ 45’ N 13$^{\circ }$ 37’ E) and Hallstätter See (47$^{\circ }$ 33’ N 13$^{\circ }$ 39’ E) were chosen as examples of glacial and oligotrophic lakes, and lake Neusiedler See (47$^{\circ }$ 56’ N 16$^{\circ }$ 50’ E) was used as a classic example of an endorheic waters.

Glacial lakes are bodies of water which formation took place due to the glacial activity, whether it be the water supplied from the glacier melting or the lake basin being carved by it (Buckel et al. 2018). Glacial lakes are most commonly oligotrophic, characterised by low levels of nutrients, low species richness and deep euphotic zone (a layer of water that can be penetrated by light).

Eutrophic lakes are the opposite of oligotrophic lakes and are characterised with high nutrient-levels, high algal content which often results in anoxia (oxygen deficit) in the bottom sediments. Both Hallstätter See and Millstätter See are classified as oligo-mesotrophic, which means their parameters fall in between oligo- and eutrophic conditions, leaning towards the former (Dokulil 1991; Ernst et al. 2009).

Millstätter See is meromictic, meaning that there are layers of water that never undergo mixing and are heavily stratified. Stratification is often enforced by differences in temperature and salinity. Conditions like this often result in the bottom layers being anoxic and uninhabited with an exception of certain organisms able to survive without oxygen, such as the *Chironomidae* (Stewart et al. 2009).

Endorheic lakes are bodies of water without an external outlet or inlet, meaning their only source of water exchange occurs via precipitation and evaporation (Soja et al. 2013). A classic example of endorheic and eutrophic lake is Neusiedlersee where the maximum depth never exceeds 1,8 m. This extreme shallowness makes the lake especially vulnerable to even minor changes in temperature or pollution (Soja et al. 2013).

Using such a variety of habitats will allow for a more versatile experimentation and testing of the biohybrid. These opposing lakes will present a different species composition and water conditions (for example, depth or light availability) which are relevant for the biohybrid development. Each of the lakes presents different challenges and enables finding new ways to overcome them which will prove useful when adapting the entity for other habitats worldwide.

### Microbial fuel cells (MFC)

**Fig. 2:**
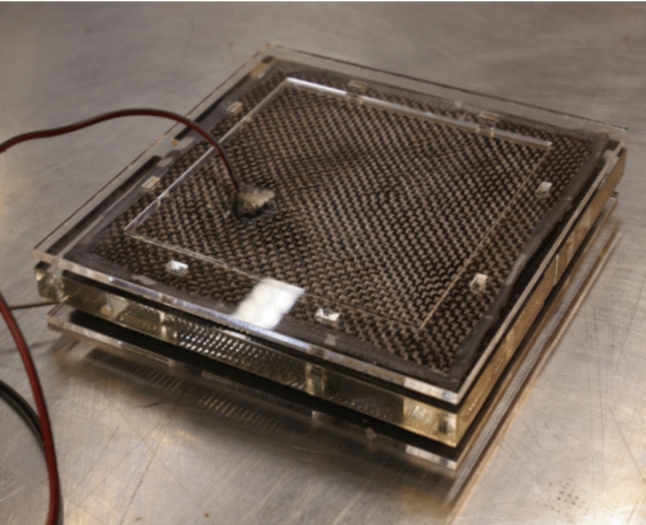
Microbial fuel cell module

Power source and energy harvesting is an important limiting factor when designing autonomous setups. In Robocoenosis, Microbial Fuel Cells (MFC) will be used as the main power source through improving their applications in the field (Fig. 2). Various projects have utilized MFCs in the non-aquatic field; however, long-term autonomy of MFC-powered biohybrids has been very limited.

**Fig. 3:**
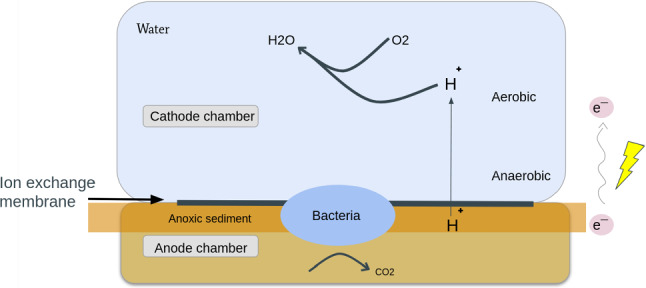
Simple schematic illustrating the working principles of the Microbial Fuel Cells. The cathode chamber is in contact with water that contains dissolved oxygen, it is also interfaced with the anode chamber via a proton exchange system that blocks oxygen but allows H$^+$ ions to pass. The anode chamber contains sediments, including bacteria and decaying matter degraded by those. This chamber has no oxygen supply, which would normally force bacteria to rely on anaerobic metabolism. However, some exoelectrogeneous bacteria (for instance *Geobacter sulfurreducens*) are able to use the more efficient aerobic metabolic pathway by transferring electrons via the electrodes present in both chambers. A chemical reaction occurs at the cathode where $\hbox {H}_{2}\hbox {O}$ is produced by the combination of oxygen with electrons and $\mathrm{H}^{+}$ ions stemming from bacterial activity in the anode chamber

MFC contain microbial life forms and use the naturally occurring organic matter as the energy source. They convert chemical energy to electrical energy by using the metabolic activity of bacteria naturally occurring in the sediments (Umaz and Wang 2019). They normally comprise of two chambers: anaerobic and aerobic, where the former is covered in the natural sediments while the latter is exposed to the oxygen present in the water (Koch et al. 2016). Simple schematic illustrating the general working principles of the MFCs is presented in Fig. 3.

MFC are one out of three most leading types of bio-energy harvesters together with enzyme-based fuel cells and biomechanical energy harvesters (Afroz et al. 2021). These and other power cells have been used as energy supply for low-power microcontrollers and other small-scale devices. A modification of traditional MFC is Benthic Microbial Fuel Cells (BMFC) that have been successfully implemented for underwater energy harvesting (Umaz et al. 2016). Aquatic environment comes with its own set of new obstacles, such as burrowing animals causing short-circuits or electrons being lost to the surrounding waters. The challenge of scaling up the BMFCs was successfully attempted by Babauta et al. (2018) with the implementation of a power management system buried in the sediment together with an anode shared by multiple cathodes. Many other alternative MFC designs have been constructed with promising results, i.e. the rise of plant-aided MFCs opened a new field of research called ”photo-reactor coupled MFCs” (Afroz et al. 2021). This dynamic subject is being consistently refined and shows encouraging results for its future large-scale implementation. Furthermore, MFCs have also been reported to operate with the microbiome of plant roots Nitisoravut and Regmi (2017) and also with algae Gajda et al. (2015). In this case, energy is indirectly obtained from photosynthesis of the associated organisms, which is complementary with the one of biomass. It is also worth noting that specific plants can be used for a phytoremediation process Kabutey et al. (2019); Guadarrama-Pérez et al. (2019), through which water is cleaned by using species that accumulate heavy metals such as lead or cadmium. The combination with MFCs may then bring the benefits of energy harvesting and bioremediation simultaneously.

**Fig. 4:**
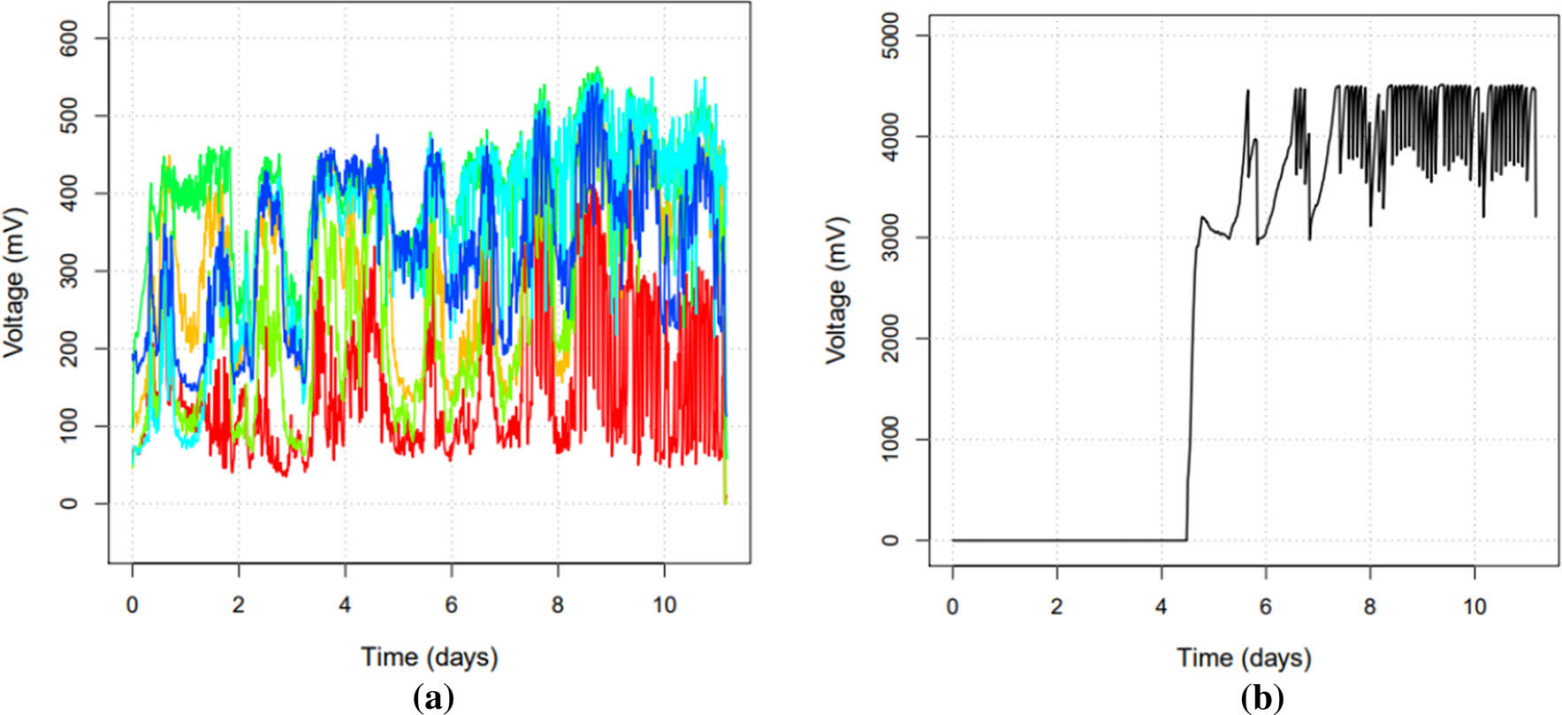
Results of experiments regarding voltage production in the prototype made for MFCs. **a** Shows voltage plotted against time for each of the six cells used in the prototype. **b** Shows voltage plotted against time for the super capacitor which is charged by the MFCs

Several challenges regarding this method have limited its use to mostly laboratory conditions until now. Dewan et al. (2008) have shown that the effects of the MFC are difficult to amplify both when attempting to use single or multiple connected cells. Recently, studies have shown promising results that using multiple stacked cells together with natural sediments generates enough power for a microcontroller, communication devices and various sensors (Santoro et al. 2017). Here, a prototype was constructed using six stacked MFCs and tested both in laboratory and in outdoor conditions in the Venice Laguna. Early experiments showed that this system is efficient enough to supply energy for the Robocoenosis low-power entity. Figure 4 presents data collected from an experiment carried out in real-world conditions a) the voltage produced by single cells individually and b) voltage produced by their collective charge supplied to the super capacitor.

In this experiment, each cell is connected to a 1F capacitor and monitored by the microcontroller. The voltage of the cells gradually rises with bacterial activity until a maximum threshold of 500 mV is reached, or when the cell voltage stabilizes, which triggers the discharge of the cell capacitor into a central 3F super-capacitor that is used to power the prototype. Note that single cells could go past the maximum threshold between monitoring events. The harvested energy is sufficient to carry out measurements and transmit data via an acoustic modem. The monitoring task is divided into a low-power sleep phase and a measurement and transmission phase. The sleep phase consumes about $10 \mu$ A, while the other phase consumes about 600 mA during 5 seconds. Once the MFC had started energy production, the prototype was able to make at least 10 measurements per day by discharging the 3F capacitor from 4.5V to 3.3V. This amounts to 140.4 J per day or 1.62 mW of power, all losses taken into account before using harvested energy.

As the voltage produced by the MFCs is directly affected by the primary production, it also gives information about the environment and the conditions in the bottom sediments. Burrowing benthic invertebrates contribute to so-called bioturbation (reworking of the sediment) which can negatively affect the proper working of the MFC (Umaz and Wang 2019). By supplying oxygen to all parts of the MFC, burrowing animals can provide oxygen to the anode and disrupt the anaerobic breathing processes.

### Biodegradability

The reduction in waste and sustainable usage of resources is one of the goals of the Robocoenosis project. Specifically, in Robocoenosis we see the possibility to reach a new level in developing biodegradable technological devices. Biodegradation is the process by which organic substances are broken down into smaller compounds by the enzymes produced by living microbial organisms. The microbial organisms transform the substance through metabolic or enzymatic processes. If this process is complete, the initial organic substances are entirely converted into simple inorganic molecules such as water, carbon dioxide and methane. Biodegradable matter is generally organic material such as plant and animal matter and other substances originating from living organisms, or artificial materials that are similar enough to plant and animal matter to be put to use by microorganisms. Commercially available bio-plastics include a diverse family of materials with differing properties.

There are three main groups:

- Bio-based (or partially bio-based), durable plastics such as bio-based polyethylene (PE), polyethylene terephthalate (PET), bio-based technical performance polymers, such as numerous polyamides (PA), or partly bio-based polyurethanes (PUR);
- Bio-based and biodegradable, compostable plastics, such as polylactic acid (PLA), polyhydroxyalkanoates (PHA), polybutylene succinate (PBS), and starch blends;
- Plastics that are based on fossil resources and are biodegradable, such as polybutylene adipate terephthalate (PBAT) and Polycaprolactone (PCL), but that may well be produced at least partly bio-based in the future.

In the project, we will investigate novel methods to accelerate or decelerate the speed of biodegradation of different types of bio-plastics or their blends, for example heating crucial sections of the outer structure in order to start the degradation process or infusing special enzymes directly into the material of the structure that can be mechanically or chemically activated when given the order.

### Lifeforms

#### Zebra mussel

**Fig. 5:**
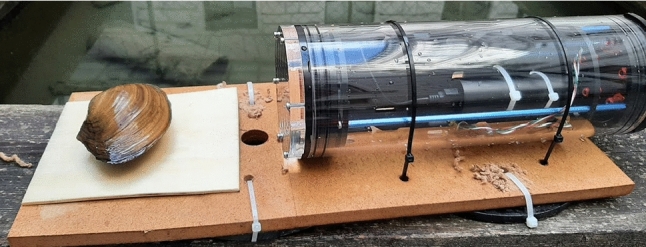
An early setup design with the use of a swan mussel *Anodonta cygnaea* mounted in front of a camera piece

**Fig. 6:**
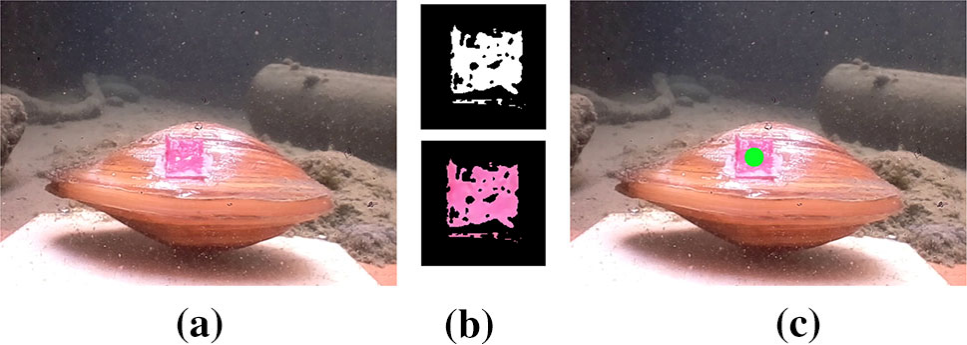
Figures show exemplary processing of images by the biohybrid entity for tracking the movement of a swan mussel. **a** An image taken by the early prototype of the biohybrid entity. **b** Extracted binary mask of from the mussel shell and the corresponding mask from the image. **c** Shell movements of the swan mussel is tracked using image analysis. The green dot at the centre of the mask shows the current position of the marker and therefore the position of the shell of the mussel

Zebra mussel (*Dreissena polymorpha*, Pallas, 1771) is one of the most widely used freshwater species for environmental monitoring. It is native to lakes of southern Russia and Ukraine, but, due to its resilience, it has become an invasive species and spread over vast areas including Europe and North America (Hoddle 2021). Mussels are good monitor species due to their longevity, large colony numbers and using filtration for feeding and breathing (Grabarkiewicz and Davis 2008). Zebra mussel’s high sensitivity to water pollution often results in abnormal burrowing behaviour or erratic valve movements. *D. polymorpha* is considered a poor oxygen-regulator and its oxygen level and temperature tolerance is narrow (Alexander and Mcmahon 2004). Although it has been found in hypoxic conditions, it can thrive only in well-oxygenated environments (Benson et al. 2021). Opening and closing of the valves have been investigated as a behavioural indicator of a potential stressor in the environment. In order to investigate mussel’s valve movements, a camera piece will be mounted on a platform (Fig. 5). Lifeforms will be attached in front of the camera using an adhesive (such as Dupla$\circledR$ PlantFix) which tested very well in other studies on glues in aquatic environments (Hartmann et al. 2016). For easily quantifiable results, a marker is added to each of the valves and using image analysis, the percentage of the opening can be calculated. This system will periodically record footage of the valves which will be analysed in an energy efficient manner. An exemplary series of images showing this process of data collection is shown in Fig. 6. The experiments resulting in the presented footage took place under real-life conditions in small ponds. The recordings were taken for 30 minutes in 5 minute intervals. They served as a method development for future experiments and using mussels as biosensors. Movement of the valves can be caused by many various factors, not all of which are a sign of pollution. For maximizing the accuracy of the monitoring experiment, it is beneficial to use multiple specimens.

#### Swan mussel

Swan mussel (*Anodonta cygnaea*, Linnaeus 1758) is one of the largest freshwater mussels, and it has been recognized as a good bioindicator due to its high sensitivity to oxygen level changes (Chojnacki et al. 2007). A high abundance of this species is a good sign of well-oxygenated conditions and nutrient-rich bottom sediments. As mentioned previously, like most other bivalves, *A. cygnea* is a good bioindicator for most heavy metals thanks to its feeding and breathing mechanism through filtration (Bilal et al. 2014). Its valve movements are significantly affected by stress, particularly low oxygen levels (Chojnacki et al. 2007). This species’ application for autonomous entities could be twofold, 1) using a similar setup to the one described previously for *D. polymorpha* or 2) base the conclusions on the presence/absence data.

**Fig. 7:**
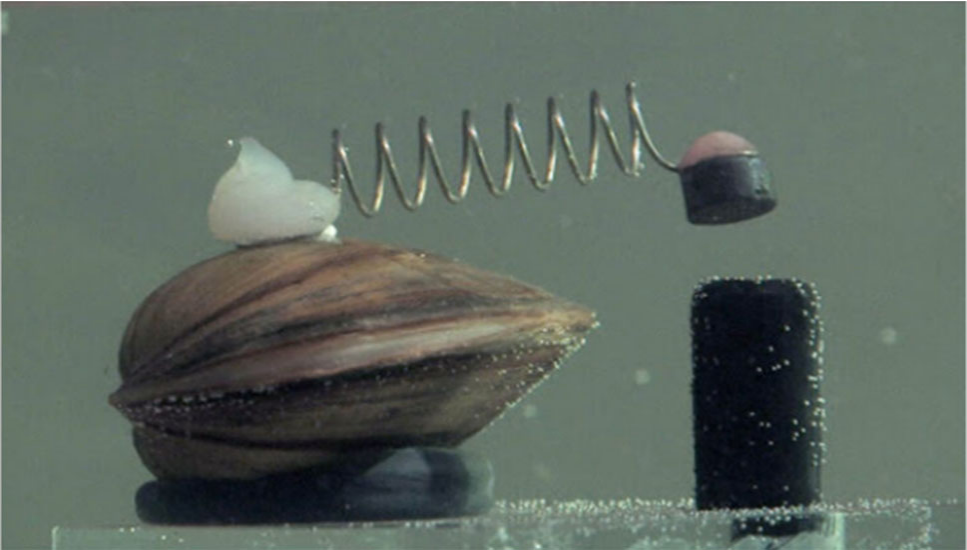
Freshwater clams used by a Polish water treatment plant. When the valves close, the sensors glued to the top valve sound the alarm and shut off the water supply. Credit: Julia Pełka (Gruba Kaáka)

Similar approach, to the one described for zebra mussel, is being used by a Polish water plant, where eight clams are connected to the system controller with a sensor attached to one valve and another in front of the mussel (Fig. 7). The alarm is triggered when sensors touch (the valves close) which is most likely a sign of stress, and thus, a presence of a pollutant (MPWiK 2019). This method can be also used for the Robocoenosis project. Special advantage of this method is its independence from the water turbidity and light presence which will prove especially useful in Neusiedlersee where the underwater visibility often reaches 0.

**Fig. 8:**
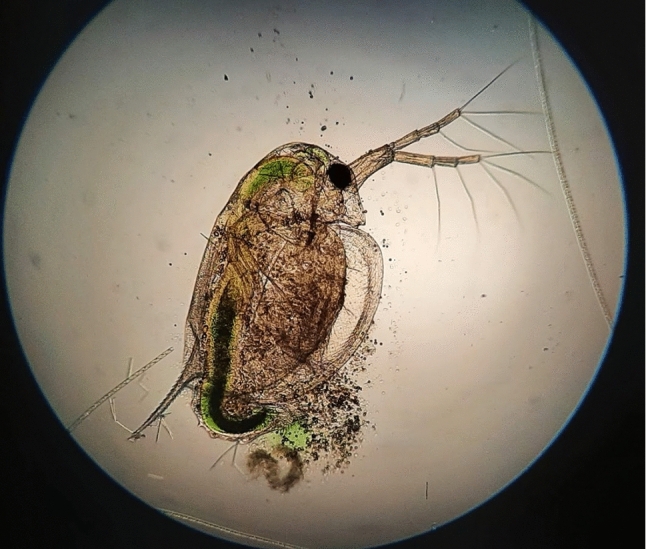
Specimen of *Daphnia* sp. under a microscope

#### Daphnia

**Fig. 9:**
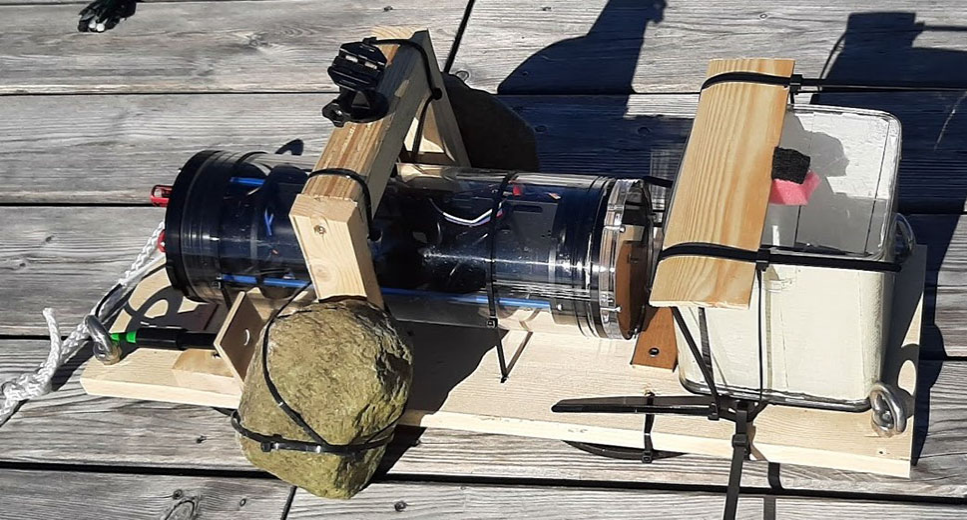
Early setup comprising of a camera monitoring the swimming patterns of *Daphnia* specimens in a flow-through system built from a shrimp breeding cage

*Daphnia* belongs to a genus *Cladocera* and has been recognised as a useful bioindicator for many years now (Fig. 8). It is very sensitive to water pollutants such as heavy metals, pesticides and other chemicals, as well as sudden temperature changes. Because of its short life cycle, easy and rapid colony growth and abundance, it is worth considering *Daphnia* (particularly *D. magna* and *D. pulex*) as one of the organisms with great potential in underwater autonomous monitoring systems. *Daphnia* is known to produce more haemoglobin to support oxygen transport in low-oxygenated environments and with rising water temperature (Ebert 2005). As the blood cells of *Daphnia* are clearly visible due to the transparent body, the increase in red oxy-haemoglobin makes the animal appear red in colour. This visual cue can be used as an indicator of poor oxygen conditions alongside other experiments taking place. These animals show phototactic behaviour (swimming towards or away from the light) which can be disturbed with the presence of pollutants and other stressors in the water. Similar disturbance can be observed in their swimming pattern, such as distribution, lurching movements and growth rate, as well as mortality during prolonged stress (Gonçalves et al. 2007). This behaviour is observable on an individual and population-level using cameras and image analysis. It is of merit to use this behaviour as a tool to measure the stress activity of specimens trapped in a flow-through container. *Daphnia* was observed remotely through a camera piece with the animals trapped in a flow-through cage (Fig. 9). Video image analysis will enable automatising the monitoring process and sounding an alarm if selected behaviours are sufficiently disrupted. Multiple early experiments took place under real-life conditions. Setup presented in Fig. 9 was lowered to approximately 1 m, and a series of videos was taken using the Raspberry Pi module programmed beforehand. Videos were taken for 30 seconds every 5 minutes to enable longer observation while remaining low-storage-friendly. Videos were taken under various light conditions including natural day light, using LED ring and underwater flashlight during nighttime.

For measuring the phototactic activity, the setup, at the time of experiment, must be shielded from the daylight and an artificial light source be turned on automatically by the robot. The ratio of *Daphnia* presenting a positive/negative phototactic behaviour will be used to calculate the phototactic index (Martins et al. 2007). This design is beneficial for Robocoenosis thanks to its simplicity of results and low energy requirements.

#### Chironomidae

**Fig. 10:**
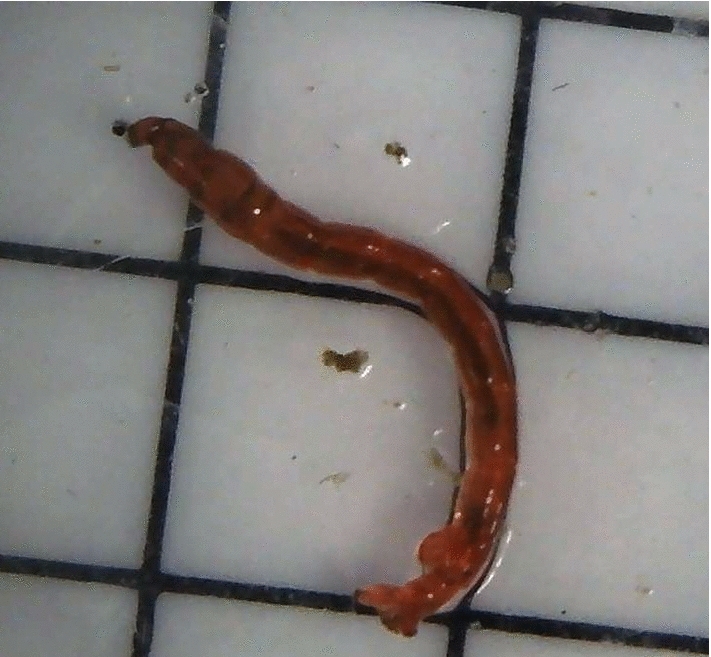
Buzzer midge larvae of *Chironomus plumosus*

*Chironomidae* is a family of insects whose larval development occurs in the water (Mclachlan 1977). The eggs of *Chironomus plumosus* (Fig. 10) are laid in the water and later, as larvae, gather the sediment using sticky silk-like substance. This results in tube-shaped structures in which the larvae reside until reaching a adult stage. *Chironomidae* contribute to, so-called, bioturbation and bioirrigation processes (Schaller 2014). Bioturbation is defined as the reworking of soils and sediments by animals or plants, and bioirrigation refers to the process by which benthic organisms flush their burrows with overlying water. The effect of bioturbation is to increase redox processes within the sediments, creating strong electric gradients that may also have a negative effect on the energy harvesting by the MFC. These larvae are relatively easy to observe thanks to their bright-red colouring and large size (around 20 mm) as well as their large densities. It has been observed that the shape and time necessary to build said tubes are good environmental indicators. The tube produced by *Chironomus plumosus* has two holes at each end, and the water is pumped by the larvae who feed by filtering out food particles from the produced stream. The community structure of the *Chironomidae* family is also a good indicator of the trophic level. The number of tolerant and intolerant species as well as species richness varies depending on the location and season (Raunio et al. 2006). Certain species (i.e. *Constempellina* sp., *Heterotanytarsus* spp., *Pagastiella* sp.) are reliable indicators of oligotrophic waters and are considered intolerant to organic pollution.

The observation of this species can be carried out using a camera. The settlement of the *C. plumosus* will be encouraged by providing a coarse surface on the camera platform or providing artificial tubes in which the larvae can settle. Another approach is placing a see-through container with sediment on the camera level so that the larvae of *Chironomidae* or other similar species can build their tubes naturally.

#### Algae

Photosynthesis is a process vital for the survival of plants, algae and certain bacteria including the cyanobacteria. Photosynthesis efficiency is often an indicator of the water quality and using it as a detection strategy for pollutants has been implemented for many years (Fai et al. 2007). There are various ways to measure the intensity of this process, and some of the most commonly used ones are measuring the oxygen production of the plant, the carbon dioxide intake, the fluorescence of the chlorophyll a, Carbon-14 tracers and others (Consalvey et al. 2005).

#### Chlorophyll *a* fluorescence

Light energy that reaches the plant can be utilized by it in three ways; (1) used to carry out the photosynthesis, (2) be expelled as heat energy or (3) emitted as light. Constant light emission by the chlorophyll (fluorescence) can be disrupted with presence of pollutants, such as herbicides. Chlorophyll *a* fluorescence is used as a rapid and reliable test in toxicological studies (Petsas and Vagi 2017). There are many ways to measure the fluorescence; however, the most commonly used one is pulse-amplitude modulation (PAM) fluorometer (Fai et al. 2007; Consalvey et al. 2005). Leeuw et al. (2013) proposed a fluorometer design that offers low-cost *in situ* measurements which could be used to detect abnormalities of the chlorophyll *a* fluorescence but also measure the water turbidity. These experiments were performed by night to eliminate the daylight impact from the measurements; however, this setup can be improved and adapted to continuous monitoring by installing the light detector inside a flow-through tube. This method can be used to calculate the photosynthesis intensity but also the algae biomass, assuming that the fluorescence intensity increases with more chlorophyll *a* and has been mostly applied for diatom studies or green algae (for example *Selenastrum capricornutum* (Consalvey et al. 2005; Fai et al. 2007). This approach opens door for more precise, continuous monitoring within the scope of the Robocoenosis project.

#### Macrophytes

A good way of determining the water quality without sample taking is examining the macrophyte community structure. It is safe to assume that after weeks of the biohybrid entity being submerged, macrophytes will start to settle around it. Identifying dominant species is a valid method of determining the lake’s trophic state and has been used as such for years (Penning et al. 2008). Apart from the dominant species, some macrophytes are a useful bioindicator just by being present in the environment. For example, *Najas flexilis* is a very rare water plant that inhabits fresh and brackish water with very low nutrient content (oligotrophic). This is because it can utilise exclusively carbon dioxide for the photosynthesis, unlike most macrophytes that can survive with using bicarbonate (the predominant form of carbon in most aquatic environments) (Wingfield et al. 2005). *N. flexilis* can be found in Millstätter See (Prochinig et al. 2014), and its presence in the lake is a good indication of its oligotrophy. Overall, freshwater macrophytes could be classified as follows (Penning et al. 2008):

1. Sensitive—only present in very clean waters and often disappear with rising eutrophication rates (i.e. *Lobelia dortmanna*),
2. Tolerant—tolerant to higher eutrophication rates (i.e. *Ceratophyllum demersum*),
3. Indifferent—present in both oligo and slightly eutrophic lakes, but tend to disappear in hypertrophic waters (i.e. *Myriophyllum spicatum*).

By observing the macrophyte community structure, a lot can be said about the lake’s nutrient content, light availability and general pollution levels. Another example of this is the use of a marine species of eelgrass *Zostera marina*. It is the most widely distributed seagrasss in the Northern Hemisphere (Krause-Jensen et al. 2005). *Z. marina* is a valuable part of marine and freshwater ecosystems, providing a substratum for benthic micro- and macro-invertebrates as well as stabilizing the bottom sediments which then reduces coastal erosion. Thanks to its high light demand, it has become a good bioindicator used through determining its maximum extension, i.e. depth limit. High levels of eutrophication lead to a higher algae abundance which reduces the light permeability and thus, the depth limit of *Z. marina* (Bertelli and Unsworth 2018). This and other algae will be used in the project Robocoenosis either by being attached to the biohybrid or by natural succession in camera view.

#### Bacterial activity

In the recent years, Microbial Fuel Cells have gained interest as not only an alternative power source, but also as a biosensor. Thanks to their working principle based on bacterial activity, MFC can provide valuable information on the biochemical oxygen demand (BOD), dissolved oxygen (DO), toxicants, microbial activity and others (Cui et al. 2019). Bacterial activity, and the electric current produced as a result, are inhibited by certain toxins or sudden environmental changes (Jiang et al. 2017). Most commonly used electricity-producing bacteria are the *Geobacter* species (especially *Geobacter sulfurreducens*) and *Shewanella oneidensis* (Poddar and Khurana 2011; Afroz et al. 2021). The concept of a self-powered biosensor opens the door to new possibilities for environmental control and will be implemented into the Robocoenosis biohybrid entity by constant monitoring the electric current and tracking its fluctuations.

The main challenges that come with using MFC as a biosensor are the long response time and their low sensitivity (Jiang et al. 2017; Zhou et al. 2017). Certain studies have focused on shortening their reaction period and have successfully achieved it by linking multiple MFCs anodes to a common cathode. Thanks to this novel approach, the constructed cell showed an immediate (up to 5 minutes) decrease in produced voltage when exposed to a sudden pH decrease, acetate or Cu$^{2+}$ presence (Jiang et al. 2017). Another challenge is the narrow range of the toxic compound levels that can be detected with an MFC-based sensor. It often occurs that the response threshold of the MFC-based biosensors exceeds the actual pollution levels (Zhou et al. 2018; Cui et al. 2019). Various studies focused on overcoming these and other challenges by modifying the MFC structure (the volume and number of chambers) and their alignment (Cui et al. 2019; Jiang et al. 2017).

#### Indices of trophic levels

Most studies using zooplanktonic and benthic microinvertebrates use the community structure (i.e. dominant species, sensitive species present) to indicate the state of the environment, rather than the behaviour of an individual (Dokulil 2003; Jeppesen et al. 2011). The presence of species typical for a certain habitat can give an idea of the trophic level of the investigated body of water. Planktonic indices have been widely used for decades (Rahkola-Sorsa 2008). According to various sources, the community structure shows relatively consistent changes with increasing eutrophication rates, with species richness declining with the rising Total Phosphorus concentration (TP). With increasing TP, the biomass of *Daphnia* declines and is replaced, to some extent, by other small cladocerans and cyclopoid copepods Jeppesen et al. (2011). This is a simplification, as the species composition changes not only with the increasing nutrients but also varies during winter/summer months and also shows changes in horizontal distribution (Rahkola-Sorsa 2008; Straile 2015). Most species of zooplankton are quite broadly distributed and are present in all types of habitats; however, there are certain species and community structures typical for certain trophy levels. Maemets (1980) created an index that clearly indicates a strong relationship between the zooplankton species composition, lake type and the trophic state, based on the present number of species of rotifers, copepods and cladocerans. The use of this and other indices can be used during the data analysis after obtaining the visual data. Observing the species composition can be managed automatically, for example through image analysis, thus controlling the tropic state of the lake can be carried out continuously.

This approach is difficult for a robot to carry out as it normally involves multiple sampling from various locations and depths with large amounts of water and sediment being tested at the same time. However, neglecting the importance of the large benthic and planktonic communities as a whole, would exclude an entire biomonitoring method group which has been a reliable trophic indicator for decades. A way to overcome this challenge is diligent observation of a small water stream, either natural or artificially created, and performing a close analysis of observed species. The most objective method is quantifying the number of present species that are characteristic for high or low trophy and reaching the conclusions by looking at the abundance of each within a certain time frame.

**Fig. 11:**
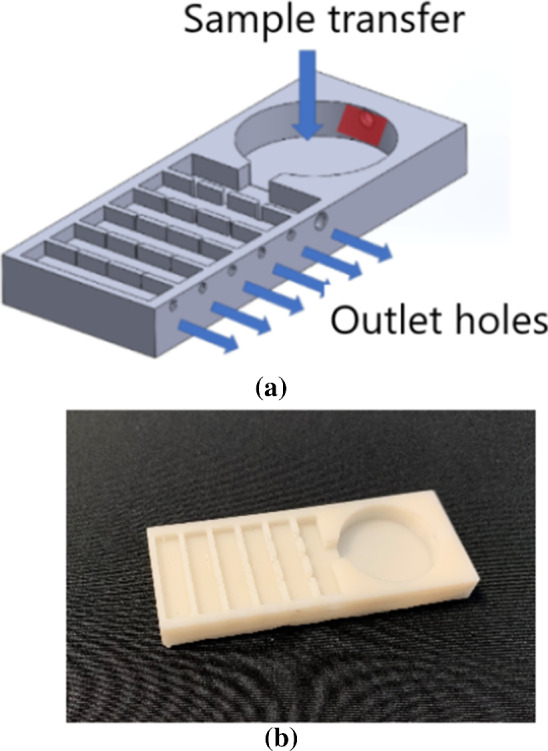
Benthic tray designed to observe benthic microinvertebrates. **a** design project and **b** 3D-printed prototype. Organisms move through the tray passing through gradually smaller tunnels which enables easier species identification

One of the methods investigating the benthic community structure with the use of underwater robotics is creating a small device, here called ”benthic tray” (Fig. 11), which will facilitate the selection of benthic organisms from the sediment and their observation under microscopic camera. This tray, 3D printed, is constructed of several channels that decrease in size gradually with a small, metallic plate heated up to >35 degrees to stress the animals and drive them inside the chamber. This setup lets the benthic microinvertebrates to move inside, sorts them by size and allows easier observation through a camera microscope. This mechanism will be tested in the laboratory and employed in the field in the future.

**Table 1:** Summary of the species and communities of interest for the Robocoenosis project

Organism(s)	Stressors	Reactions to stressors	Ways of observation
*Dreissena polymorpha*	Temperature, pesticides, herbicides, oxygen, predation	Valve closing, mortality	Image analysis, magnetic sensor
*Anodonta cygnaea*	Temperature, pesticides, herbicides, oxygen	Valve closing, mortality	Image analysis, magnetic sensor
*Daphnia*	Temperature, pollution, salinity, pH, heavy metals	Swimming behaviour, excessive sinking, lower fertility, slower growth, disruption in phototactic ability	Image analysis
*Chironomidae*	Trophy, organic pollution	Community structure, tube building abilities	Image analysis
Microinvertebrates	pH, ammonia, phosphate, conductivity, temperature	Presence/absence data, dominant species, community structure	Continuous observation through a benthic tray or glass tubes
Algae and macrophytes	Pesticides, herbicides, light availability	Disruption in fluorescence, presence/absence, growth rate, discoloration	Fluorometry, image analysis

## Discussion

In this paper, we have outlined various novel ways to use lifeforms in biohybrid entities for environmental monitoring (Table 1). We show that living organisms have potential to become a promising alternative for “traditional” sensors. Furthermore, the biohybrid entity will be using low-power electronics and energy harvesting performed by the MFCs that have been recognised as a feasible energy source for underwater robotics. Both of these concepts need further development and will be investigated within the scope of this project. We have introduced an ambitious concept of a “lifeform in a loop” which have shown encouraging results in certain recent projects (Schmickl et al. 2013; Wahby et al. 2018; Komasilova et al. 2020). The main features of Robocoenosis, such as “long term autonomy” and “bio-degradability”, will open many doors for future environmental monitoring. It will provide the opportunity for continuous control with minimal impact on the environment. Some concepts of Robocoenosis might prove useful for other fields such as classical robotics, passive monitoring and others (Albaladejo et al. 2010). Using biohybrids as autonomous, biohybrid entities is a step towards a more efficient, less invasive and more balanced environmental monitoring.

First initiatives of biomonitoring with the use of lifeforms have been attempted in the past. MOSSELMONITOR$\textregistered$ (AquaDect 2021) performs the water quality assessment by placing two sensors on each valve of a mussel and continuously measuring the distance between them. It extracts data on the water quality by measuring the strength of electric signal between the valves and sounds an alarm if certain thresholds are passed. Similar setup is being used in Poland by various water treatment facilities where eight clams are constantly exposed to the water stream. Valve movements of two or more specimens are again, a signal of a pollutant present in the water (MPWiK 2019). These setups, while giving promising results, are limited to using bivalves for biomonitoring and are powered by batteries or electricity, which this project aims to substitute with an autonomous power-source. Additionally, Robocoenosis tries to ensure the biodegradability of the monitoring structure and developing an entity as close to completely noninvasive as possible.

Another example of using lifeforms as biomonitors is Daphnia Toximeter which uses video image analysis to investigate the behaviour of *Daphnia* sp. and trigger an alarm should the behaviour be disrupted (Enviro-Analytical 2021). This device tracks their swimming pattern, growth ratio, distribution and other behaviours in a continuous stream of water. This promising application of *Daphnia* is, however, heavily laboratory-based and, just like the MOSSELMONITOR$\textregistered$ is material and energy intensive. The setup developed by the Robocoenosis aims for the miniaturization of computational devices which will enable real-time monitoring of bodies of water while being energy-efficient, autonomous and completely field-based.

Results based on behaviour monitoring are less parameter-specific than regular sampling and *ex situ* investigations. The approach proposed by Robocoenosis will be unlikely to provide data on a specific parameter and so, for that classical sensors are still advised. Organisms respond to the overall state of the environment which is why it is crucial to pick species as bioindicators with an incredibly thorough understanding of their biology. To overcome this challenge, we will combine the use of the well-described species with less common ones that we might get a chance to observe, as well as basic sensors, to gather a well-rounded data on the state of the environment. However, this also causes a certain ambiguity in the obtained results, particularly when a behaviour is affected by a multitude of factors. For example, the valve movements of *D. polymorpha* can be affected by various ecological, as well as environmental, factors (Dzierzyńska-Białończyk et al. 2019). The mussel closes its shells in order to protect its inner parts in response to various signs of danger. It appears to be strongly induced by the irritation of the shell or fish kairomones (presence of a predator). Mussels also respond to chemical queues released by injured conspecifics (i.e. crushed mussels). This challenge can be overcome by a thorough data analysis and observing whether the valve movements are a response to a chronic or acute stress.

The major challenge that comes with the use of planktonic organisms, like *Daphnia* is the need to entrap them while allowing the water to flow freely. To solve this issue, the Robocoenosis setup will consist of a flow-through chamber to allow the organisms to come in contact with the pollutant and be able to catch the food particles but also isolated enough, so they remain protected from the predators. The survival and reproductive efficiency of *Daphnia* has been studied extensively and is strongly linked with the water physical and chemical parameters and so, it is an excellent example of an organism that can be used for biomonitoring with a biohybrid entity.

Biodegradability, reduction in waste and sustainable usage of resources, is a general issue in modern society. In Robocoenosis, we see the possibility to reach a new level in developing biodegradable technological devices, especially due the already high degree of biological matter included in the setup design. The use of lifeforms as surrogates for “artificial” sensors will minimize the use of non-biodegradable parts and thus enable the major parts of the robot to remain in the environment and completely decompose (see Sect. 2.4).

Ethically, the project Robocoenosis will use exclusively invertebrates and so, it does not fall into the scope of animal rights laws. However, we will continue to be respectful of all lifeforms and eliminate or minimize any potential stress they may experience throughout the experiments with the help of camera monitoring and other noninvasive methods.

In this paper, we have presented first early stages of developing a biohybrid entity. Many of the results to be obtained regarding the use of lifeforms, low-power electronics and biodegradable materials represent the huge potential that noninvasive environmental monitoring has in any future projects worldwide. We presented early results and the possibilities of the underwater robotics in context of environmental monitoring together with the use of lifeforms as biological sensors.
